# How to Give Iron

**Published:** 1887-08

**Authors:** 


### How to Give Iron.

Professor Germain See does not know everything, even if he does live in Paris. Concerning the administration of iron he says: "It will precipitate the gastric juice taken before meals, therefore take it while there is something in the stomach to prevent this. It is not known how it gets into the circulation, because it is not seen to go out. In any case, give it *with meals.*" The latter sentence shows his ignorance. Most people eat food containing tanic acid; tanic acid unites with iron to form an insoluble tannate of iron. One hour after eating, the tanic acid will have passed from the stomach; then the iron should be given, and those who have heretofore been disappointed in the use of iron will be surprised at the beneficial results obtainable from the agent.—*Index.*

